# Fractal–fractional model for analysis of tuberculosis infection using Ethiopia incidence data

**DOI:** 10.1002/qub2.70045

**Published:** 2026-07-14

**Authors:** Kumama Regassa Cheneke, Fatmawati Fatmawati, Mustafa Bayram

**Affiliations:** ^1^ Department of Mathematics Hawassa College of Teacher Education Hawassa Ethiopia; ^2^ Department of Mathematics, Faculty of Science and Technology Airlangga University Surabaya Indonesia; ^3^ Department of Computer Engineering Biruni University Istanbul Türkiye

**Keywords:** convolution theorem, Euler fractional method, fixed point theory, fractional‐order, Laplace transform, numerical simulation, tuberculosis

## Abstract

In this study, a fractional‐order $SEI_{u}ITR$ model was proposed to examine the transmission of tuberculosis (TB) in the community. The proposed model was rigorously examined for well‐posedness. The basic reproduction number was also derived, and the disease‐free equilibrium was obtained. The local and global stabilities of the proposed model were also examined. The proposed model was fitted with the actual TB incidence in Ethiopia, while numerical simulations were carried out in MATLAB to validate the results obtained in this study. Local and global sensitivity analyses were carried out to determine the key parameters that influence the transmission of TB in the community. Phase‐plane portraits and phase‐plane diagrams demonstrate the dynamic process of the state variables. The results indicate that as the fractional‐order increases, the number of infections decreases due to the memory effect of early detection and treatment, while as the order decreases, the number of infections increases. Early detection and treatment have a profound effect on recovery and prevalence.

## 1 INTRODUCTION

Tuberculosis (TB) is one of the most dangerous infectious diseases caused by the bacterium *Mycobacterium tuberculosis* [1, 2, 3, 4, 5]. One of the most important features of TB is its latent form, where people who have been in contact with the bacteria or are carriers may not even know their status.

The primary infection stage of TB is when the immune cells in the body recognize and fight off the invading bacteria. In some instances, the immune system is able to destroy the bacteria entirely [6, 7]. However, some of the bacteria may survive and live inside the body. One of the biggest problems in the fight against TB is that many people infected with the disease do not show symptoms during the early stages of infection. When symptoms do appear, they are usually mild and may include influenza‐like symptoms such as low‐grade fever, tiredness, and coughing. After the primary infection, a person may develop latent TB infection, where the immune system is able to hold off the bacteria by creating a barrier in the lungs. During this period, the bacteria remain alive but inactive, and no clinical symptoms are observed. Active TB develops when the immune system fails to suppress the infection, allowing the bacteria to proliferate and cause disease in the lungs or other parts of the body.

Active TB may occur shortly after initial infection or emerge months or even years later due to reactivation of latent infection. The symptoms of active TB typically worsen gradually and may include persistent cough, hemoptysis, chest pain, fever, chills, night sweats, loss of weight, loss of appetite, fatigue, and general malaise [8, 9].

The symptoms of this type of TB depend on the organs that are affected and may include the kidneys, liver, central nervous system, heart, lymph nodes, bones and joints, skin, blood vessels, and larynx. The symptoms include fever, night sweats, loss of weight, fatigue, pain, and loss of organ function. In children, especially infants, the symptoms of TB can be very different from those of adults. In adolescents, the symptoms may resemble those of adults, while in younger children, the symptoms may include fever, failure to gain weight, developmental delays, and neurological problems due to TB meningitis [7, 8].

Mathematical modeling is a key component in the study of complex phenomena in the real world by formulating them in a mathematical framework [10]. In the field of epidemiology, mathematical models have been extensively employed to describe the dynamics of disease transmission and assess the efficacy of control measures [11]. Traditional integer‐order dynamical systems have been employed to model TB transmission, including the role of exogenous reinfection and endogenous reactivation of recovered or pseudo‐recovered individuals [3, 12]. Nevertheless, these models have limitations in describing the memory and hereditary properties of TB, which is associated with long latent periods and delayed treatment responses.

Fractional‐order models have been recognized as useful tools for implementing memory effects and have been extensively employed to improve the accuracy and realism of infectious disease models [13, 14, 15]. Specifically, the non‐singular memory kernels of the Caputo–Fabrizio (CF) and Atangana–Baleanu–Caputo (ABC) operators make it possible to implement memory effects without encountering singularities, which is advantageous for modeling long‐term dynamics of infectious diseases.

The fractional‐order α measures the intensity of memory activation by incorporating the entire history of disease evolution into the current state of the system. Lower values of *α* indicate higher memory effects, which represent important epidemiological processes such as disease latency, waning immunity, and pathogen reactivation [16, 17]. These properties are particularly important in the case of TB, in which treatment compliance and late diagnosis are key factors in the persistence of the disease.

Recent research has emphasized the importance of awareness, reinfection, and effective intervention strategies in controlling TB transmission [9, 12, 13, 16, 17, 18, 19, 20, 21, 22]. However, TB still constitutes a major health problem worldwide, especially in developing nations. It is in light of these challenges that this paper proposes a CF fractional TB model that includes both informed and uninformed compartments to explore the effect of memory activation on TB transmission based on incidence data from Ethiopia.

## 2 RESULTS

### 2.1 Non‐negativity of solutions

In this section, we prove that all state variables of the system are non‐negative under the fractional operator.

Theorem 1 (Non‐negativity of solutions). The solutions of the TB system of Equation (118) with anticipated initial conditions behave non‐negative for all t≥0.

The proof is based on the generalized mean value theorem for the CF fractional derivative, which states that if a function reaches a minimum at a point, then the fractional derivative of the function at that point is non‐positive.

First to show the non‐negativity of S(t), suppose that there is a first time $t_{1}>0$ such that

(1) $$S\left(t_{1}\right)=0,S\left(t\right)>\;0\;\text{for}\;0\leq t<t_{1}.$$

For system of Equation (118),

(2) Dtα0CFSt1=Λ>0.

However, since S(t) attains its minimum at $t_{1}$, the generalized fractional mean value theorem implies

(3) Dtα0CFSt1<0,

which is a contradiction. Thus,

(4) S(t)≥0,∀t≥0.

Again to show the non‐negativity of E(t) assume there exists $t_{2}$ such that

(5) $$E\left(t_{2}\right)=0.$$

Then,

(6) Dtα0CFEt2=βSt2Iut2+θIt2≥0.

Thus, E(t) cannot become negative.

To show the non‐negativity of $I_{u}\left(t_{3}\right)$, if $I_{u}\left(t_{3}\right)=0$, then

(7) Dtα0CFIut3=κEt3≥0.

Thus,

(8) $$I_{u}\left(t\right)\geq 0.$$

Again to show the non‐negativity of I(t), if $I\left(t_{4}\right)=0$, then

(9) Dtα0CFIt4=ϕIut4+ωEt4≥0.

Thus,

(10) I(t)≥0.

Also, to show the non‐negativity of T(t), if $T\left(t_{5}\right)=0$, then

(11) Dtα0CFTt5=ρIt5≥0.

Thus,

(12) T(t)≥0.

To show the non‐negativity of R(t), if $R\left(t_{6}\right)=0$, then

(13) Dtα0CFRt6=ηTt6≥0.

Gives *R*(*t*) ≥ 0 and satisfies the non‐negativity condition.

Therefore, the solutions remain biologically feasible and this completes the proof.

### 2.2 Invariant region using Mittag‐Leffler function

Theorem 2 *(Invariant region). Consider system of Equation (* *118* *) with non‐negative initial conditions. Then the region*

(14) $$\Omega =\left\{\left(S,E,I_{u},I,T,R\right)\in R_{+}^{6}:0\leq N\left(t\right)\leq \frac{\Lambda }{\mu }\right\},$$

*is positively invariant*.

Summing left and right of system of Equation (118), we obtain

(15) Dtα0CFN(t)=Λ−μN(t).

Since $I_{u},I,T\geq 0$ by non‐negativity. Consider the auxiliary scalar equation

(16) Dtα0CFX(t)=Λ−μX(t),X(t),X(0)=N(0)≥0.

Applying the Laplace transform to Equation (16):

(17) LDtα0CFX(t)=sX‾(s)−N(0)(1−α)s+α=Λs−μX‾(s).

Solving for $\bar{X}\left(s\right)$:

(18) $$\bar{X}\left(s\right)=\frac{N\left(0\right)+\Lambda \left(1-\alpha \right)/\mu }{s\left(1+\mu \left(1-\alpha \right)\right)+\mu \alpha }+\frac{\alpha \Lambda /s}{s\left(1+\mu \left(1-\alpha \right)\right)+\mu \alpha }.$$

Using partial fraction decomposition and the Laplace transform of the Mittag‐Leffler function, the inverse transform gives

(19) $$X\left(t\right)=\frac{\Lambda }{\mu }+\left(N\left(0\right)-\frac{\Lambda }{\mu }\right)\;E_{1}\left(-\frac{\mu \alpha }{1+\mu \left(1-\alpha \right)}t\right),$$

where $E_{1}\left(-x\right)$ is the Mittag‐Leffler function of order one. Since $0<E_{1}\left(-x\right)\leq 1$ for x≥0, we have

(20) $$0\leq X\left(t\right)\leq \frac{\Lambda }{\mu },\forall t\geq 0.$$

By the fractional comparison principle, since

(21) Dtα0CFN(t)≤Dtα0CFX(t),N(0)=X(0).

It follows that

(22) $$N\left(t\right)\leq X\left(t\right)\leq \frac{\Lambda }{\mu },\forall t\geq 0.$$

Hence, the region

(23) $$\Omega =\left\{\left(S,E,I_{u},I,T,R\right)\in R_{+}^{6}:0\leq N\left(t\right)\leq \frac{\Lambda }{\mu }\right\},$$

is positively invariant.

### 2.3 Existence and uniqueness of solutions

One of the known conditions for the well‐posedness of the model is the existence and uniqueness of the solution. Consider the CF fractional epidemiological system of Equation (118) with non‐negative initial conditions. The existence and uniqueness of the solutions can be guaranteed by the Banach fixed‐point theorem.

Theorem 3 *(Existence and uniqueness). Let*

(24) $$S\left(0\right),E\left(0\right),I_{u}\left(0\right),I\left(0\right),T\left(0\right),R\left(0\right)\geq 0,$$

*and assume all model parameters are non‐negative constants. Then there exists a unique solution*

(25) $$\left(S\left(t\right),E\left(t\right),I_{u}\left(t\right),T\left(t\right),R\left(t\right)\right)\in C\left[0,\tau \right],R_{+}^{6},$$

*for some* τ>0.

We write system of Equation (118) in the abstract form

(26) Dtα0CFX(t)=F(t,X(t)),X(0)=X0,

where

(27) $$X\left(t\right)=\left(\begin{array}{c} S\left(t\right)\\ E\left(t\right)\\ I_{u}\left(t\right)\\ I\left(t\right)\\ T\left(t\right)\\ R\left(t\right) \end{array}\right),X_{0}=\left(\begin{array}{c} S\left(0\right)\\ E\left(0\right)\\ I_{u}\left(0\right)\\ I\left(0\right)\\ T\left(0\right)\\ R\left(0\right) \end{array}\right),$$

and ***F*** is the vector of right‐hand side functions of the system of Equation (118). By definition of the CF integral, this system of equations can be re‐written in the equivalent integral equation

(28) $$z\left(t\right)=z_{0}+\frac{1-\alpha }{\alpha }\int_{0}^{t}F\left(\tau ,z\left(\tau \right)\right)\;d\tau .$$

Define an operator or function ϰ on the Banach space

(29) $$B=\left\{z\in C\left(\left[0,\tau \right],R_{+}^{6}\right)\;{\left\|z\right\|}_{\infty }=\underset{t\in \left[0,\tau \right]}{\sup}\left\|z\right\|<\infty \right\},$$

by

(30) $$\left(\varkappa Y\right)\left(t\right)=Y_{0}+\frac{1-\alpha }{\alpha }\int_{0}^{t}F\left(\tau ,Y\left(\tau \right)\right)\;d\tau .$$

Since F is Lipschitz continuous with respect to z in any bounded subset of $R_{+}^{6}$, there exists a constant L>0 such that

(31) $$\left\|F\left(t,Y_{1}\left(t\right)\right)-\left(F\left(t,Y_{2}\left(t\right)\right)\right)\right\|\;\leq \;L\left\|Y_{1}\left(t\right)-Y_{2}\left(t\right)\right\|,\forall t\in \left[0,\tau \right].$$

Consequently, for all $Y_{1},Y_{2}\in B$, we have

$$\left\|\left(\varkappa Y_{1}\right)\left(t\right)-\left(\varkappa Y_{2}\right)\left(t\right)\right\|\;\leq \;\frac{1-\alpha }{\alpha }\int_{0}^{t}\left\|F\left(\tau ,Y_{1}\left(\tau \right)\right)-\;\left(F\left(\tau ,Y_{2}\left(\tau \right)\right)\right)\right\|d\tau$$

(32) $$\leq \frac{1-\alpha }{\alpha }L\int_{0}^{t}\left\|Y_{1}\left(\tau \right)-Y_{2}\left(\tau \right)\right\|d\tau ,$$

(33) $$\leq \frac{1-\alpha }{\alpha }L\tau {\left\|Y_{1}-Y_{2}\right\|}_{\infty },$$

by choosing appropriately, that τ>0

(34) $$K=\frac{1-\alpha }{\alpha }L\tau <1,$$

the operator ϰ is a contraction operator on B. According to the Banach fixed point theorem, a contraction operator on a complete metric space has a unique fixed point. Therefore, the contraction operator ϰ on B possesses a distinct fixed point $z^{*}\in B$ satisfying

(35) $$z^{*}=\varkappa z^{*}.$$

This fixed point corresponds to the unique solution to the system of Equation (118) on the interval [0,t].

### 2.4 Disease‐free equilibrium

The disease‐free equilibrium (DFE) ($X_{0}$) represents a state where there is no disease in the population [14, 15, 18, 23, 24, 25, 26, 27]. This means that the populations in the exposed and infectious classes are zero, that is, $E=I_{u}=I=T=0$. At equilibrium, the CF fractional derivatives vanish. Setting

(36) Dtα0CFS=Dtα0CFE=Dtα0CFIu=Dtα0CFI=Dtα0CFT=Dtα0CFR=0.

We obtain the algebraic system of equation

(37) 0=Λ−μS,0=−μR.

Therefore, the solution of the preceding system of equation yields

(38) $$X_{0}=\left(S,E,I_{u},I,T,R\right)=\left(\frac{\Lambda }{\mu },0,0,0,0,0\right).$$

### 2.5 Basic reproduction number

In a completely susceptible population, the expected number of secondary infections caused by one infected person is defined as the basic reproduction number $R_{0}$, which is calculated as the spectral radius of the next generation matrix [28, 29, 30, 31, 32, 33, 34]. For the model, let’s consider F the matrix of the new infection terms and V the transition matrix:

(39) $$F=\left(\begin{array}{c} \beta S\left(I_{u}+\theta I\right)\\ 0\\ 0\\ 0 \end{array}\right),V=\left(\begin{array}{c} \left(\omega +\kappa +\mu \right)E\\ -\kappa E+\left(\phi +\delta +\mu \right)I_{u}\\ -\phi I_{u}-\omega E+\left(\rho +\delta +\mu \right)I\\ -\rho I+\left(\eta +\delta +\mu \right)T \end{array}\right).$$

The Jacobian matrices of vectors f and v are the matrices F and V respectively and given by

(40) $$F=\left(\begin{array}{cccc} 0 & \beta S & \beta \theta S & 0\\ 0 & 0 & 0 & 0\\ 0 & 0 & 0 & 0\\ 0 & 0 & 0 & 0 \end{array}\right),V=\left(\begin{array}{cccc} \omega +\kappa +\mu & 0 & 0 & 0\\ -\kappa & \phi +\delta +\mu & 0 & 0\\ -\omega & -\phi & \rho +\delta +\mu & 0\\ 0 & 0 & -\rho & \eta +\delta +\mu \end{array}\right).$$

Computation of $R_{0}=FV^{-1}$ gives

(41) $$R_{0}=\frac{\beta S_{0}}{\omega +\kappa +\mu }\left(\frac{\kappa }{\phi +\delta +\mu }+\frac{\phi }{\rho +\delta +\mu }+\frac{\omega }{\rho +\delta +\mu }\right).$$

In the $R_{0}$, substituting $S_{0}$ with $\frac{\Lambda }{\mu }$ gives,

(42) $$R_{0}=\frac{\beta \Lambda }{\mu \left(\omega +\kappa +\mu \right)}\left(\frac{\kappa }{\phi +\delta +\mu }+\frac{\phi }{\rho +\delta +\mu }+\frac{\omega }{\rho +\delta +\mu }\right).$$

This formula expresses the expected number of secondary infections in a completely susceptible population. If $R_{0}<1$, the infection is not sustained and will die out. If $R_{0}>1$, then the disease can invade the population.

### 2.6 Endemic equilibrium in terms of $R_{0}$

In the state of equilibrium, the CF fractional derivatives are zero:

(43) Dtα0CFS=Dtα0CFE=Dtα0CFIu=Dtα0CFI=Dtα0CFT=Dtα0CFR=0.

Setting the right‐hand sides of the system of Equation (118) to zero, we have the following algebraic system of equations:

(44) $$\begin{array}{ll} 0= & \Lambda -\beta S\left(I_{u}+\theta I\right)-\mu S,\\ 0= & \beta S\left(I_{u}+\theta I\right)-\left(\omega +\kappa +\mu \right)E,\\ 0= & \kappa E-\left(\phi +\delta +\mu \right)I_{u},\\ 0= & \phi I_{u}+\omega E-\left(\rho +\delta +\mu \right)I,\\ 0= & \rho I-\left(\eta +\delta +\mu \right)T,\\ 0= & \eta T-\mu R. \end{array}$$

The last three equations of Equation (118) can be expressed in terms of the variable *E* as

(45) $$I_{u}=\frac{\kappa }{\phi +\delta +\mu }E,I=\frac{\phi I_{u}+\omega E}{\rho +\delta +\mu }=\frac{\phi \kappa +\omega \left(\phi +\delta +\mu \right)}{\left(\phi +\delta +\mu \right)\left(\rho +\delta +\mu \right)}E,T=\frac{\rho }{\eta +\delta +\mu }I,R=\frac{\eta }{\mu }T.$$

Substituting these expressions for variables $I_{u}$ and I into the first two equations of Equation (118), we get

(46) $$\beta S\left(I_{u}+\theta I\right)=\left(\omega +\kappa +\mu \right)E.$$

Using the expression for $R_{0}$:

(47) $$R_{0}=\frac{\beta S_{0}}{\omega +\kappa +\mu }\left(\frac{\kappa }{\phi +\delta +\mu }+\frac{\phi }{\rho +\delta +\mu }+\frac{\omega }{\rho +\delta +\mu }\right),S_{0}=\frac{\Lambda }{\mu },$$

the susceptible population at endemic equilibrium, it can be written as

(48) $$S=\frac{S_{0}}{R_{0}}.$$

Thus, the endemic equilibrium $\left(X_{e}\right)$ is given by

(49) $$X_{e}=\left(S,E,I_{u},I,T,R\right)=\left(\frac{S_{0}}{R_{0}},E,\frac{\kappa }{\phi +\delta +\mu }E,\frac{\phi \kappa +\omega \left(\phi +\delta +\mu \right)}{\left(\phi +\delta +\mu \right)\left(\rho +\delta +\mu \right)}E,\frac{\rho }{\eta +\delta +\mu }I,\frac{\eta }{\mu }T\right),$$

where *E* can be computed from

(50) $$E=\frac{\mu \left(R_{0}-1\right)S_{0}}{\omega +\kappa +\mu +\beta \left(\frac{\kappa }{\phi +\delta +\mu }+\theta \frac{\phi \kappa +\omega \left(\phi +\delta +\mu \right)}{\left(\phi +\delta +\mu \right)\left(\rho +\delta +\mu \right)}\right)S_{0}}.$$

### 2.7 Local stability of equilibria

Theorem 4 *(Local stability of TB‐free steady state). Let* $X_{0}=\left(S,E,I_{u},I,T,R\right)=\left(S_{0},0,0,0,0,0\right)$ *the DFE with* $S_{0}=\frac{\Lambda }{\mu }$ *. Then,*

- *If* $R_{0}<1$ *, then the DFE is locally asymptotically stable.*
- *If* $R_{0}>1$ *, then the DFE is unstable.*

Let $X_{I}=\left(\begin{array}{c} E\\ I_{u}\\ I\\ T \end{array}\right)$ the TB‐infected compartments and we construct the Lyapunov function,

(51) $$V=E+I_{u}+I+T,$$

which is non‐negative and has an equilibrium value of 0 at the DFE. We calculate the CF fractional derivative of *V* with respect to time *t*

(52) Dtα0CFV=Dtα0CFE+Dtα0CFIu+Dtα0CFI+Dtα0CFT.

By substituting the system equations and evaluating at $S\leq S_{0}$, we can write that

(53) Dtα0CFV≤(ω+κ+μ)R0−1E≤0ifR0<1.

By applying the standard Lyapunov theory for fractional‐order systems, we can conclude that, since Dtα0CFV≤0 and V is positive definite, the DFE is locally asymptotically stable for $R_{0}<1$, and unstable if $R_{0}>1$.

Theorem 5 (*Local stability of the endemic equilibrium*). *Let* $X_{e}$ *be the endemic equilibrium expressed in terms of* $R_{0}>1$ *. Then the endemic equilibrium is locally asymptotically stable.*

Let the deviation from the endemic equilibrium be given by

(54) $$\overset{ˇ}{X}=X-X_{e}.$$

Define Lyapunov function to show the local stability state

(55) $$V=\sum_{i}{\overset{ˇ}{X}}_{i}-{\overset{ˇ}{X}}_{i}\ln\frac{{\overset{ˇ}{X}}_{i\;}+X_{e,i}}{X_{e,i}},$$

where the sum is over all compartments $i=S,E,I_{u},I,T,R$. This function is positive definite and vanishes at $X=X_{e}$. Differentiating along the trajectories of system Equation (118) and using the relation between compartments at the endemic equilibrium expressed in terms of $R_{0}$, we obtain

(56) Dtα0CFV≤0,

equality if and only if $\overset{ˇ}{X}=0$. For fractional‐order systems, the endemic equilibrium $X_{e}$ is thus locally asymptotically stable whenever $R_{0}>1$, according to Lyapunov’s direct technique.

### 2.8 Global stability of the DFE

Theorem 6 (*Global stability of DFE*). *If* $X_{e}=\left(S,E,I_{u},I,T,R\right)=\left(S_{0},0,0,0,0,0\right)$ *is the DFE with* $S_{0}=\frac{\Lambda }{\mu }$ *, then the TB‐free steady state is globally asymptotically stable in the feasible region of*

(57) $$\Omega =\left\{\left(S,E,I_{u},I,T,R\right)\in R_{+}^{6}:S+E+I_{u}+I+T+R\leq S_{0}\right\},$$

the basic reproduction number $R_{0}\leq 1$.

To study the global behavior of the infection, we focus on the infected population given by the vector

(58) $$X_{I}=\left(\begin{array}{c} E\\ I_{u}\\ I\\ T \end{array}\right).$$

To prove the stability, we define the Lyapunov function

(59) $$V=E+I_{u}+I+T.$$

This function is positive definite in the feasible region Ω and only attains zero at the DFE when $E=I_{u}=I=T=0$.

Taking the CF fractional derivative with respect to time *t* along the solutions of the system of equations, we obtain

(60) Dtα0CFV=Dtα0CFE+Dtα0CFIu+Dtα0CFI+Dtα0CFT.

Replacing the system equations for the infected population, we have

(61) Dtα0CFV=βSIu+θI−(ω+κ+μ)E−(φ+δ+μ)Iu−(ρ+δ+μ)I−(η+δ+μ)T≤βS0Iu+θI−(ω+κ+μ)E−(φ+δ+μ)Iu−(ρ+δ+μ)I−(η+δ+μ)T,

where we have used the fact that $S\leq S_{0}$ in the feasible region. Using the definition of the basic reproduction number,

(62) $$R_{0}=\frac{\beta S_{0}}{\omega +\kappa +\mu }\left(\frac{\kappa }{\phi +\delta +\mu }+\frac{\phi }{\rho +\delta +\mu }+\frac{\omega }{\rho +\delta +\mu }\right),$$

we obtain

(63) Dtα0CFV≤(ω+κ+μ)R0−1E.

Thus, when $R_{0}\leq 1$, we have Dtα0CFV≤0 and equality occurs only when $E=I_{u}=I=T=0$, that is, at the DFE.

Using LaSalle’s invariance principle for fractional systems, all trajectories in Ω will converge to the largest invariant set where Dtα0CFV=0, which is the DFE.

The global asymptotic stability of the DFE $X_{0}$ is guaranteed if $R_{0}\leq 1$.

### 2.9 Global stability of TB model endemic equilibrium

Theorem 7 *If the assumption* $R_{0}\geq 1$ *holds, then the TB model endemic equilibrium*

(64) $$X_{e}=\left(S_{e},E_{e},I_{u,e},I_{e},T_{e},R_{e}\right),$$

*of the TB dynamic transmission fractional‐order system with CF fractional derivative guarantees a globally asymptotically stable behavior in the feasible region*

(65) $$Ω=\left\{\left(S,E,I_{u},I,T,R\right)\in R_{+}^{6}\right\}.$$

In order to show the guarantee of the global stability of the system, we consider the following Lyapunov function

(66) $$V=\left(S-S_{e}-S_{e}\;\ln\frac{S}{S_{e}}\right)+\left(E-E_{e}-E_{e}\;\ln\frac{E}{E_{e}}\right)+\left(I_{u}-I_{u,e}-I_{u,e}\;\ln\frac{I_{u}}{I_{u,e}}\right)+\left(I-I_{e}-I_{e}\;\ln\frac{I}{I_{e}}\right)+\left(T-T_{e}-T_{e}\;\ln\frac{T}{T_{e}}\right)+\left(R-R_{e}-R_{e}\;\ln\frac{R}{R_{e}}\right).$$

Accordingly, the function satisfies,

(67) V≥0,

and

(68) $$V=0,\text{if}\;\text{and}\;\text{only}\;\text{if}\;X=X_{e}.$$

Since the function

(69) $$f\left(x\right)=x-x_{e}-x_{e}\;\ln\left(\frac{x}{x_{e}}\right),$$

is non‐negative for all *x* > 0.

Applying the CF fractional derivative along system trajectories,

(70) Dtα0CFV=1−SeSDtα0CFS+1−EeEDtα0CFE+1−Iu,eIuDtα0CFIu+1−IeIDtα0CFI+1−TeTDtα0CFT+1−ReRDtα0CFR.

On the other hand, from the equation,

(71) Dtα0CFE=βSIu+θI−(ω+κ+μ)E.

At equilibrium,

(72) $$\beta S_{e}\left(I_{u,e}+\theta I_{e}\right)=\left(\omega +\kappa +\mu \right)E_{e}.$$

Thus,

(73) 1−EeEDtα0CFE≤0,

with equality holds if and only if

(74) $$E=E_{e}.$$

Again, for $I_{u}$,

(75) Dtα0CFIu=κE−(ϕ+δ+μ)Iu,

and using

(76) $$\kappa E_{e}=\left(\phi +\delta +\mu \right)I_{u,e}.$$

Hence,

(77) 1−Iu,eIuDtα0CFIu≤0.

Also for I,

(78) Dtα0CFI=ϕIu+ωE−(ρ+δ+μ)I,

and

(79) $$\phi I_{u,e}+\omega E_{e}=\left(\rho +\delta +\mu \right)I_{e}.$$

Thus,

(80) 1−IeIDtα0CFI≤0.

Also for T,

(81) Dtα0CFT=ρI−(η+δ+μ)T,

and

(82) $$\rho I_{e}=\left(\eta +\delta +\mu \right)T_{e},$$

thus,

(83) 1−TeTDtα0CFT≤0.

But,

(84) Dtα0CFS=Λ−βSIu+θI−μS.

Considering the equilibrium condition,

(85) $$\Lambda =\beta S_{e}\left(I_{u,e}+\theta I_{e}\right)+\mu S_{e},$$

yields,

(86) 1−SeSDtα0CFS≤0.

Again,

(87) Dtα0CFR=ηT−μR,

and

(88) $$\eta T_{e}=\mu R_{e}.$$

Thus,

(89) 1−ReRDtα0CFR≤0.

Summing all components yields,

(90) Dtα0CFV≤0,

with equality holds if and only if

(91) $$\left(S,E,I_{u},I,T,R\right)=\left(S_{e},E_{e},I_{u,e},I_{e},T_{e},R_{e}\right).$$

Therefore, by LaSalle’s invariance principle for fractional‐order systems, the endemic equilibrium is globally asymptotically stable whenever

(92) $$R_{0}>1.$$

### 2.10 Numerical scheme of model

The fractional TB model was solved numerically using the fractional Euler method adapted for the CF fractional derivative. This approach is well‐suited for epidemiological models incorporating memory effects, as it accounts for past states of the system, providing a more realistic representation of disease progression [35, 36, 37].

Let the fractional‐order be α∈(0,1]. Consider the following general fractional differential equation:

(93) Dtα0CFX(t)=F(t,X(t)),X(0)=X0.

Using a fixed step size *h*, the CF fractional derivative can be approximated by the numerical fractional Euler scheme that is simple and suitable for the analysis of various epidemiological models [35, 36]:

(94) $$X_{n+1}=X_{n}+\frac{\left(1-\alpha \right)}{M\left(\alpha \right)}F\left(t_{n},X_{n}\right)+\frac{\alpha h}{M\left(\alpha \right)}F\left(t_{n},X_{n}\right),$$

where M(α) is the normalization function of the derivative. This method was iteratively applied to the entire compartments of the TB model, including $S,E,I_{u},I,T,$ and R. This method also guarantees the positivity and feasibility of the solution variables. The method was implemented in MATLAB to efficiently simulate the fractional system. The fractional weight α offers flexibility to interpolate between memoryless behavior (α=1) and memory‐dependent behavior (α<1), in order to match long‐term epidemiological data.

### 2.11 Consistency, stability, and convergence of the fractional Euler scheme

In this subsection, we examine the numerical properties of the CF fractional Euler scheme for the TB model. Let the fractional TB model be written in compact form as

(95) Dtα0CFX(t)=F(t,X(t)),X(0)=X0,

where $X\left(t\right)=\left(S,E,I_{u},I,T,R\right)$ and F represents the nonlinear transmission dynamics. The CF fractional Euler scheme is given by

(96) $$X_{n+1}=X_{n}+\frac{1-\alpha }{M\left(\alpha \right)}F\left(t_{n},X_{n}\right)+\frac{\alpha h}{M\left(\alpha \right)}\sum_{k=0}^{n}F\left(t_{k},X_{k}\right),$$

where *h* is the time step and M(α) is the normalization function. Let’s say the function F meets the Lipschitz condition:

(97) $$\left\|F\left(t,X_{1}\right)-F\left(t,X_{2}\right)\right\|\leq L\left\|X_{1}-X_{2}\right\|.$$

#### 2.11.1 Consistency

Theorem 8 *The Caputo*–*Fabrizio fractional Euler method is consistent with the Caputo*–*Fabrizio TB model, and the local truncation error* $\left(\tau_{n}\right)$ *meets the requirements of*

(98) $$\tau_{n}=O\left(h\right).$$

Consistency is established by comparing the exact solution with the numerical scheme. Using Taylor expansion of the exact solution,

(99) $$X\left(t_{n+1}\right)=X\left(t_{n}\right)+hX^{\prime }\left(t_{n}\right)+O\left(h^{2}\right).$$

From the definition of the CF fractional derivative,

(100) $$X^{\prime }\left(t_{n}\right)=F\left(t_{n},X_{n}\right).$$

Substituting into the Taylor expansion gives

(101) $$X\left(t_{n+1}\right)=X\left(t_{n}\right)+hF\left(t_{n},X_{n}\right)+O\left(h^{2}\right).$$

The numerical scheme gives

(102) $$X_{n+1}=X_{n}+hF\left(t_{n},X_{n}\right)+O\left(h\right).$$

Thus, the local truncation error is

(103) $$\tau_{n}=X\left(t_{n+1}\right)-X_{n+1}=O\left(h\right).$$

Thus, the method is consistent of order one.

#### 2.11.2 Stability

Theorem 9 *If the function F satisfies the Lipschitz condition*

(104) $$\left\|F\left(t,X_{1}\right)-F\left(t,X_{2}\right)\right\|\leq L\left\|X_{1}-X_{2}\right\|,$$

*then the CF fractional Euler scheme is stable for sufficiently small step size h*.

Assume that two numerical solutions with different initial conditions are represented by $X_{n}$ and ${\tilde{X}}_{n}$. Following that, the description of an error is

(105) $$e_{n}=X_{n}-{\tilde{X}}_{n}.$$

From the numerical scheme,

(106) $$e_{n+1}=e_{n}+\frac{1-\alpha }{M\left(\alpha \right)}\left[F\left(t_{n},X_{n}\right)-F\left(t_{n},{\tilde{X}}_{n}\right)\right]+\frac{\alpha h}{M\left(\alpha \right)}\sum_{k=0}^{n}\left[F\left(t_{k},X_{k}\right)-F\left(t_{k},{\tilde{X}}_{k}\right)\right].$$

Taking norms and using Lipschitz condition,

(107) $$\left\|e_{n+1}\right\|\leq \left(1+Ch\right)\left\|e_{n}\right\|.$$

Applying this inequality recursively,

(108) $$\left\|e_{n}\right\|\leq \left\|e_{0}\right\|e^{Ct_{n}}.$$

Thus, the numerical solution remains bounded and the scheme is stable.

#### 2.11.3 Convergence

Theorem 10 *The Caputo–Fabrizio fractional Euler scheme converges to the exact solution of the TB model, and the global error satisfies*

(109) $$\max_{0\leq n\leq N}\left\|X\left(t_{n}\right)-X_{n}\right\|=O\left(h\right).$$

Define the global error

(110) $$E_{n}=X\left(t_{n}\right)-X_{n}.$$

Using consistency,

(111) $$E_{n+1}=E_{n}+h\left[F\left(t_{n},X\left(t_{n}\right)\right)-F\left(t_{n},X_{n}\right)\right]+O\left(h^{2}\right).$$

Using Lipschitz condition,

(112) $$\left\|E_{n+1}\right\|=\left(1+Lh\right)\left\|E_{n}\right\|+Ch^{2}.$$

Applying discrete Gronwall inequality,

(113) $$\left\|E_{n}\right\|\leq Ch.$$

Thus,

(114) $$\lim_{h\to 0}\left\|X\left(t_{n}\right)-X_{n}\right\|=0.$$

Therefore, the fractional Euler method converges to the exact solution.

### 2.12 Curve fitting and estimation of the parameters

To verify the effectiveness of the presented $SEI_{u}ITR$ fractional model, the TB incidence data for Ethiopia from 2000 to 2023 were utilized [38]. The parameters of the model were determined by fitting the model’s incidence curve to the real data using the MATLAB programming language. The fitting was conducted by minimizing the difference between the TB incidence data and the model’s output using a least squares method. The goodness of fit of the model was determined by computing the root mean square error, which is given by

(115) $$\text{RMSE}=\sqrt{\frac{1}{n}\sum_{i=1}^{n}{\left(I_{\text{model}}\left(t_{i}\right)-I_{\text{data}}\left(t_{i}\right)\right)}^{2},}$$

where $I_{\text{model}}\left(t_{i}\right)$ is the TB incidence predicted by the model at time $t_{i}$, $I_{\text{data}}\left(t_{i}\right)$ is the real TB incidence data at time $t_{i}$, and n is the total number of data points. Figure 1 illustrates the comparison of the observed TB incidence and the result obtained from the model simulation. The results obtained demonstrate a good comparison and thus verify the model’s ability to simulate the TB transmission in the Ethiopian population. The estimation of the parameters ensures reliable quantitative estimates of the model’s key parameters associated with TB transmission and intervention strategies. The estimated model is then employed in the sensitivity analysis, phase‐plane analysis, and numerical simulations. The estimated model is then employed in projecting the TB incidence in the next 10 years under existing intervention strategies and thus emphasizes the need for enhanced intervention strategies to reduce TB prevalence in the region.

**FIGURE 1:**
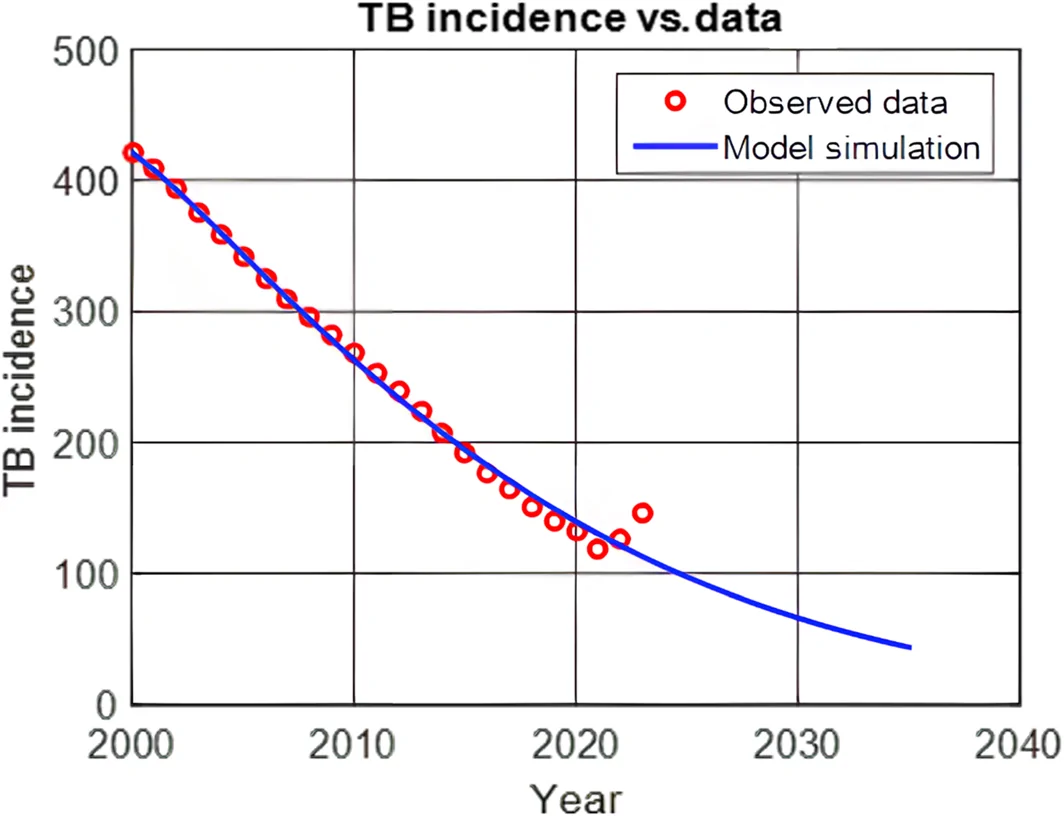
Fitted TB model to the incidence data. TB, tuberculosis.

### 2.13 Sensitivity analysis on a local and global level

#### 2.13.1 Local sensitivity analysis

Local sensitivity analysis is a measure of how a small change in each parameter affects the model [39]. The normalized forward sensitivity index for a parameter *p* with respect to *R*_0_ is given by

(116) $$\Upsilon_{p}^{R_{0}}=\frac{{\partial R}_{0}}{\partial p}\times \frac{p}{R_{0}}.$$

Table 1 describes model parameters, values, and sources used in the TB transmission model. Estimated parameters were obtained by fitting the model to TB incidence data, while fixed parameters were adopted from standard epidemiological literature.

**TABLE 1:** Model parameters, values and sources.

Parameter	Value	Source
β	0.000001/year	Assumed, estimated through model fitting
μ	0.014290/year	Based on average life expectancy [40]
θ	0.640239/year	Estimated (model fitting)
ω	0.071320/year	Estimated (model fitting)
κ	0.159446/year	Estimated (model fitting)
ϕ	0.126341/year	Estimated (model fitting)
δ	0.043744/year	Estimated (model fitting)
ρ	0.173612/year	Estimated (model fitting)
η	0.227914/year	Estimated (model fitting)
Λ	28.909128/year	Estimated (model fitting)

Local sensitivity indices of the basic reproduction number of $R_{0}$ are described in Table 2.

**TABLE 2:** Normalized sensitivity indices description.

Parameter	Value	Normalized sensitivity index
β	0.000001/year	1.0000
μ	0.014290/year	−0.4652
θ	0.640239/year	0.2285
ω	0.071320/year	−0.7924
κ	0.159446/year	0.5641
ϕ	0.126341/year	−0.0138
δ	0.043744/year	−0.2927
ρ	0.173612/year	−0.2285
η	0.227914/year	−0.3120
Λ	28.909128/year	1.0000

Global sensitivity analysis partial rank correlation coefficients (PRCC) of the basic reproduction number $R_{0}$ with respect to model parameters is described in Table 3. Baseline values are taken from MATLAB estimation, and parameter ranges are used for Latin hypercube sampling (LHS).

**TABLE 3:** Global sensitivity indices description.

Parameter	Baseline	LB	UB	PRCC
β	0.000001/year	0.000008	0.000012	0.95
μ	0.014290/year	0.0114	0.0171	−0.46
θ	0.640239/year	0.5000	0.8000	0.22
ω	0.071320/year	0.0500	0.1000	−0.78
κ	0.159446/year	0.0800	0.2500	0.55
ϕ	0.126341/year	0.0800	0.1500	−0.01
δ	0.043744/year	0.03	0.06	−0.29
ρ	0.173612/year	0.12	0.25	−0.23
η	0.227914/year	0.15	0.30	−0.33
Λ	28.909128/year	20	40	0.94

Abbreviations: LB, lower bound; PRCC, partial rank correlation coefficient; UB, upper bound.

Figure 2 shows a plot of the local sensitivity indices for all parameters in the model. From the results, it is clear that the transmission rate parameter β and the modification parameter θ are directly proportional to $R_{0}$, implying that a small change in these parameters results in a significant increase in disease transmission. Conversely, the parameter ω associated with interventions is highly negative, which shows that it is a key parameter in controlling $R_{0}$.

**FIGURE 2:**
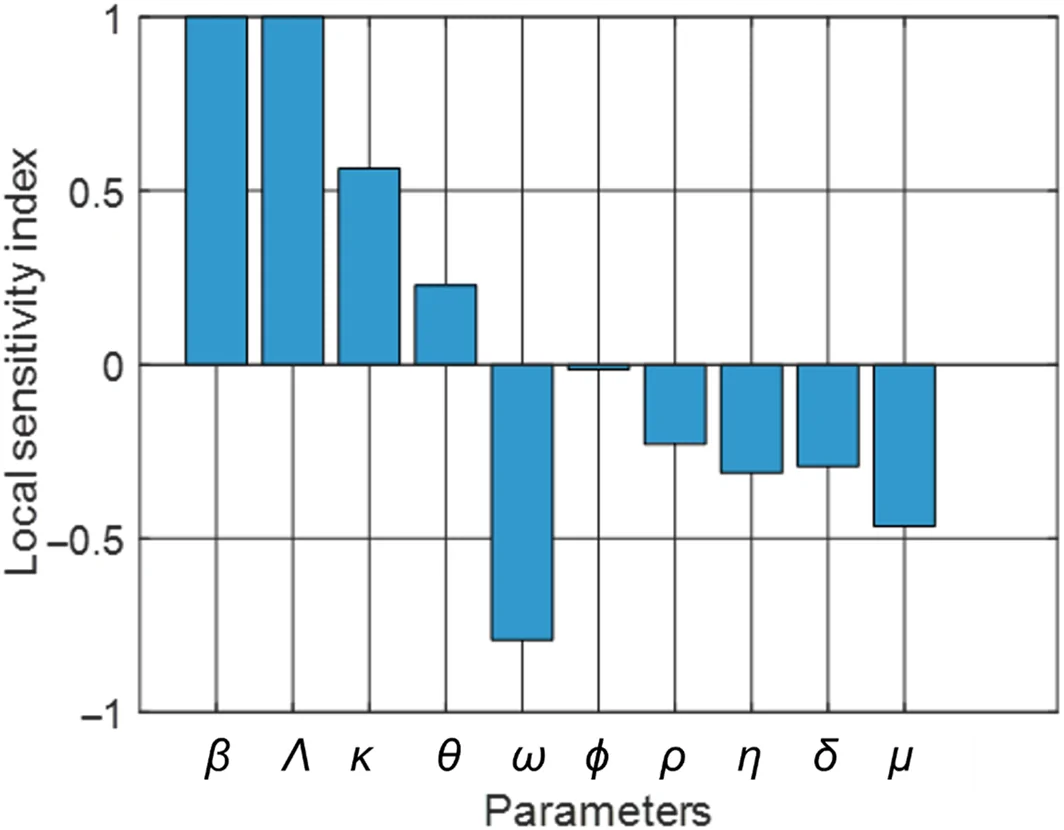
Local sensitivity analysis simulation.

#### 2.13.2 Global sensitivity analysis

On the other hand, global sensitivity analysis is used to investigate the overall influence of each parameter across its entire range, considering possible interactions between the parameters. To obtain the global sensitivity indices, the LHS method with PRCC is used. Figure 3 shows the results for the global sensitivity analysis. From the results, it is evident that *β* and *θ* are the most important parameters in increasing *R*_0_, while *ω* is the most sensitive parameter in reducing $R_{0}$. This shows that control strategies focusing on reducing transmission and changing behavior are important in controlling the spread of TB. From the above discussions, it is evident that the results from the local and global sensitivity analyses are important in controlling the spread of TB in a public health intervention context.

**FIGURE 3:**
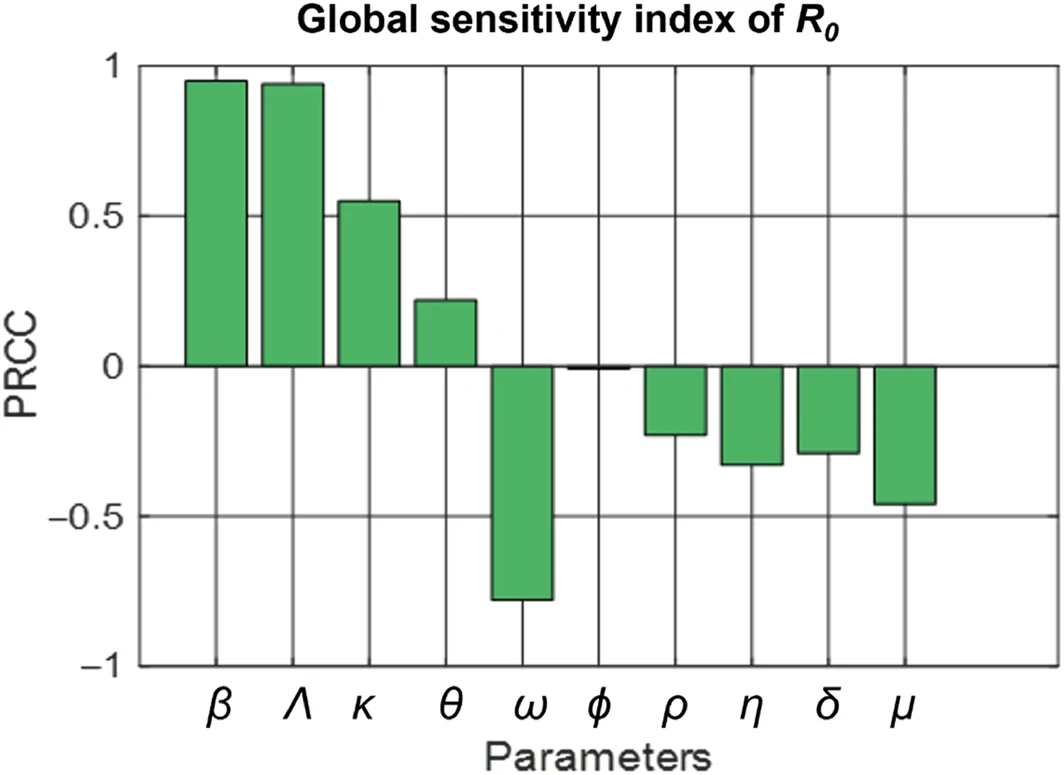
Global sensitivity analysis simulation. PRCC, partial rank correlation coefficient.

### 2.14 Numerical simulations of the population dynamics

The numerical simulations of the population dynamics are described in the subsequent figures (Figures 4, 5, 6, 7, 8, 9, 10, 11, 12, 13, 14, 15). Moreover, the initial conditions and time of simulations are visible on the figures.

**FIGURE 4:**
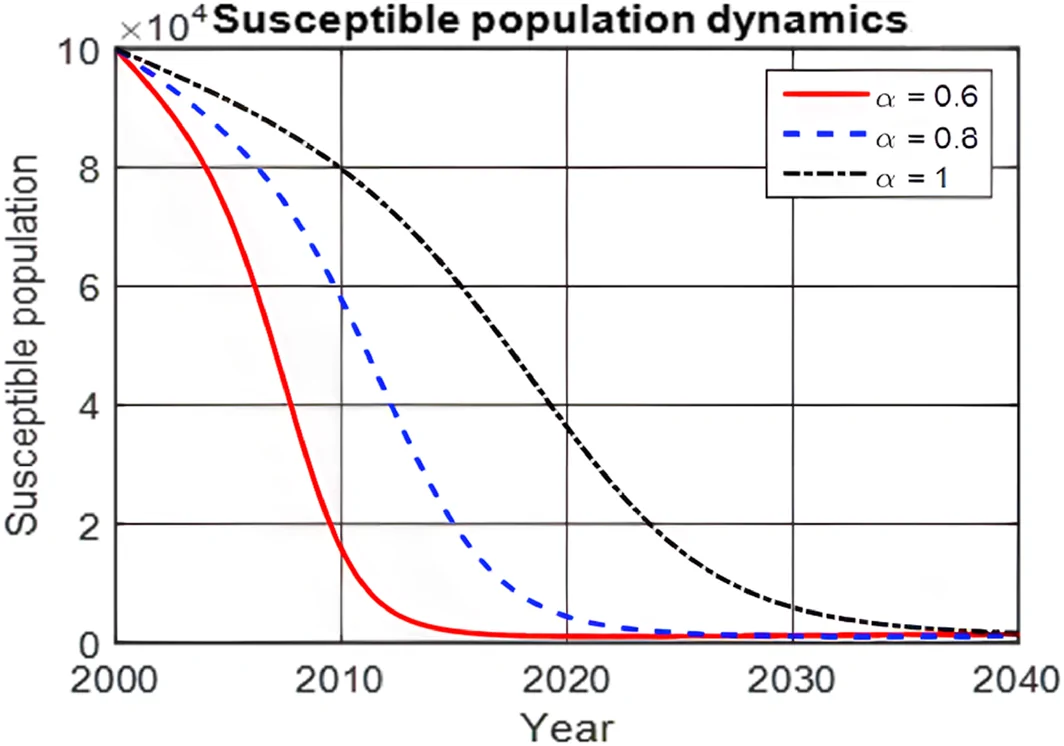
Susceptible population dynamics for different fractional orders.

**FIGURE 5:**
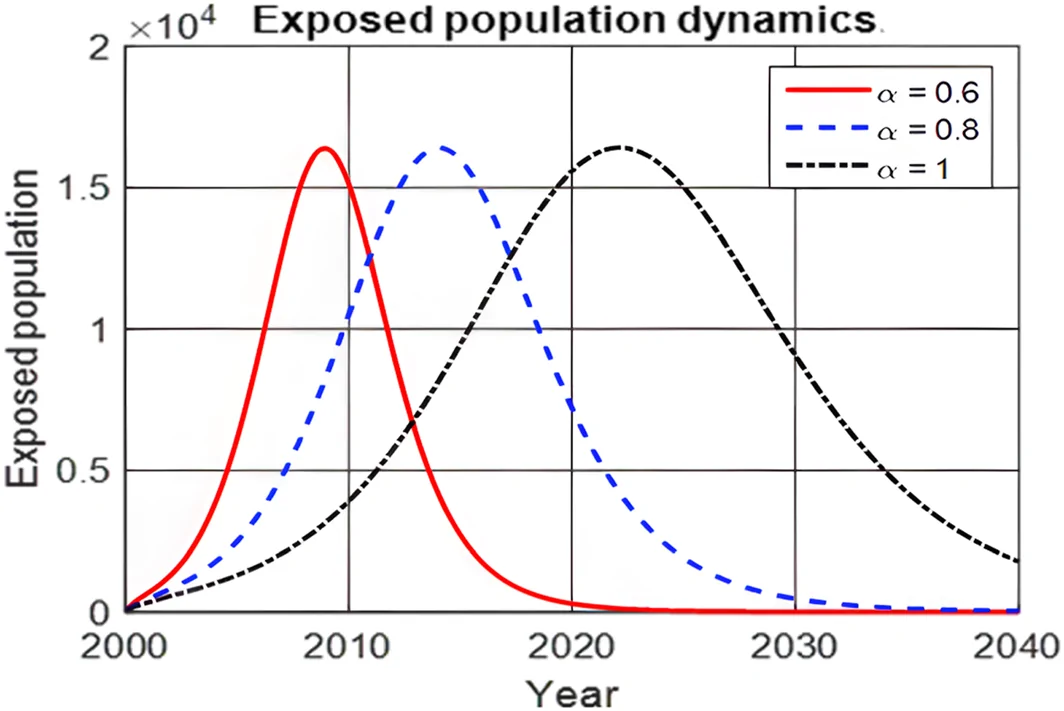
Exposed population dynamics for different fractional orders.

**FIGURE 6:**
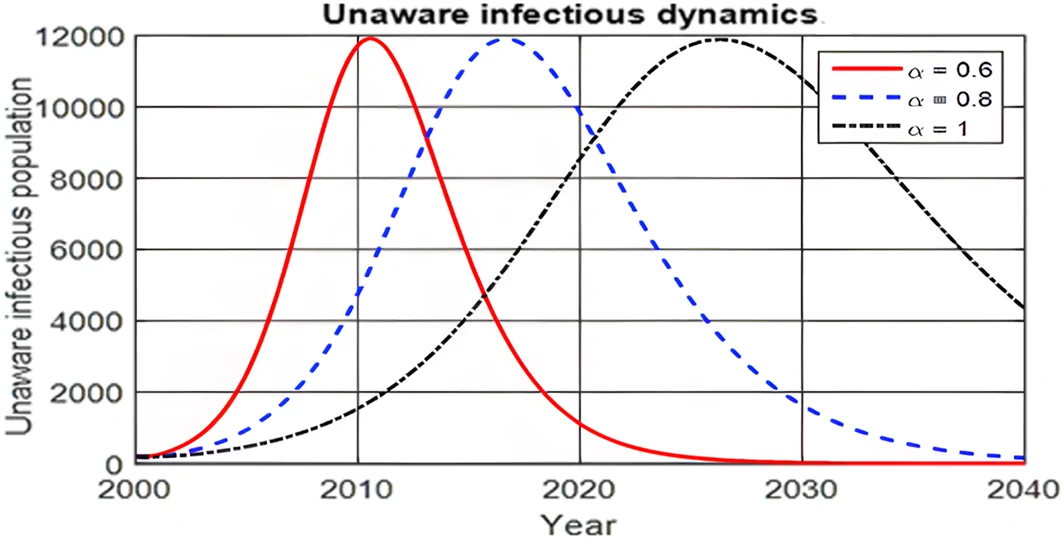
Unaware infectious dynamics for different fractional orders.

**FIGURE 7:**
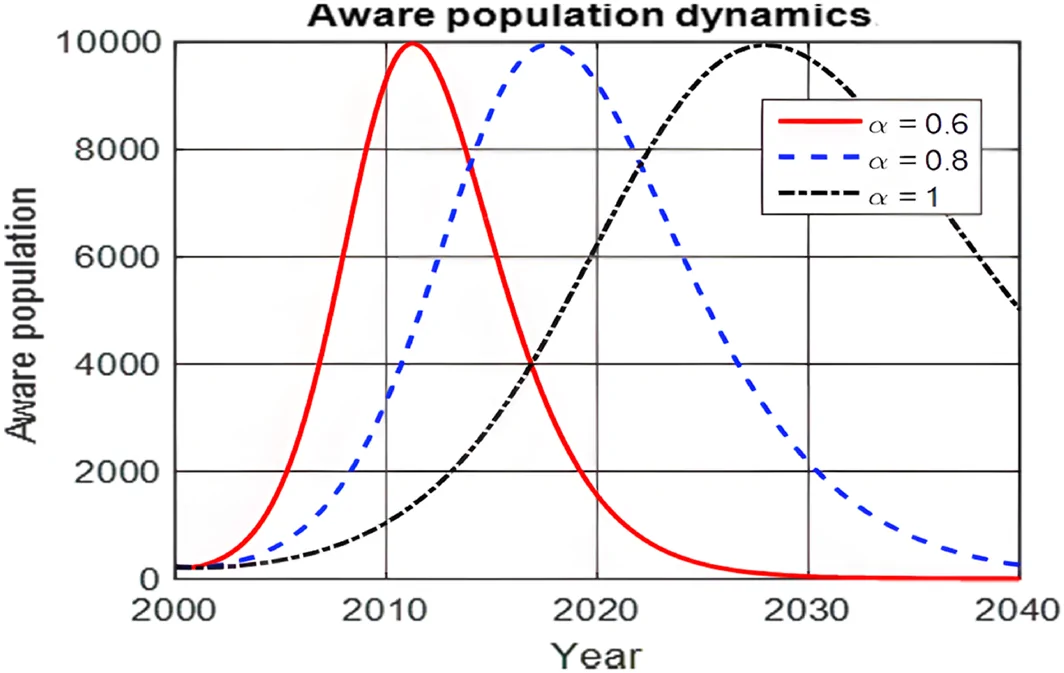
Aware population dynamics for different fractional orders.

**FIGURE 8:**
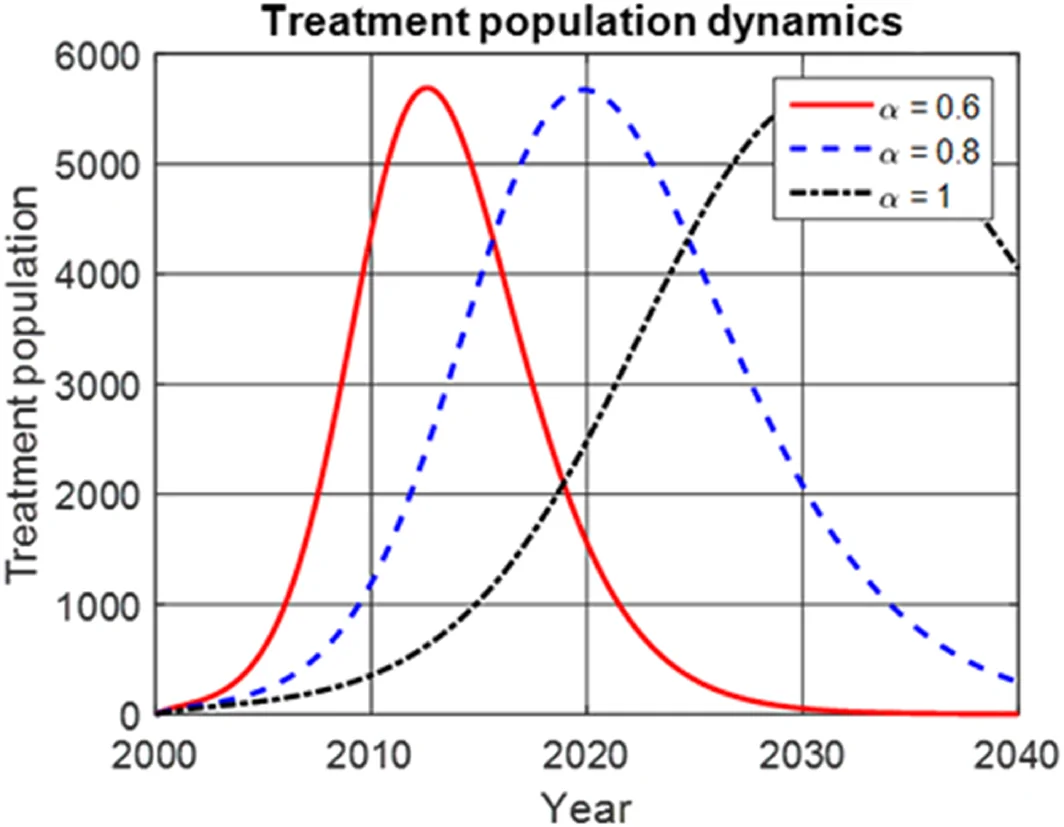
Treatment population dynamics for different fractional orders.

**FIGURE 9:**
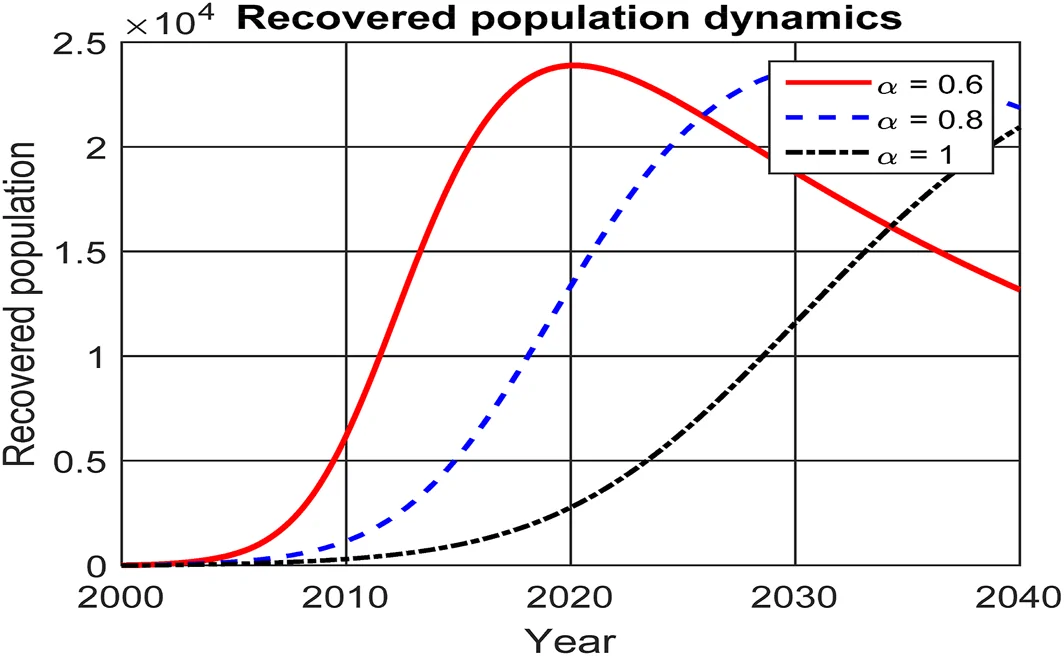
Recovered population dynamics for different fractional orders.

**FIGURE 10:**
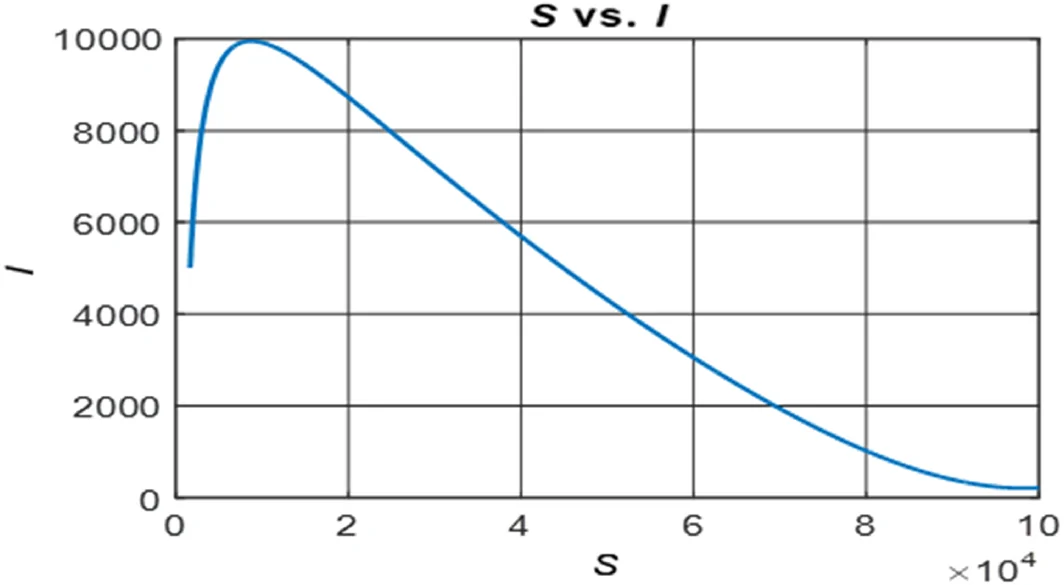
Phase plane plot of susceptible versus aware population.

**FIGURE 11:**
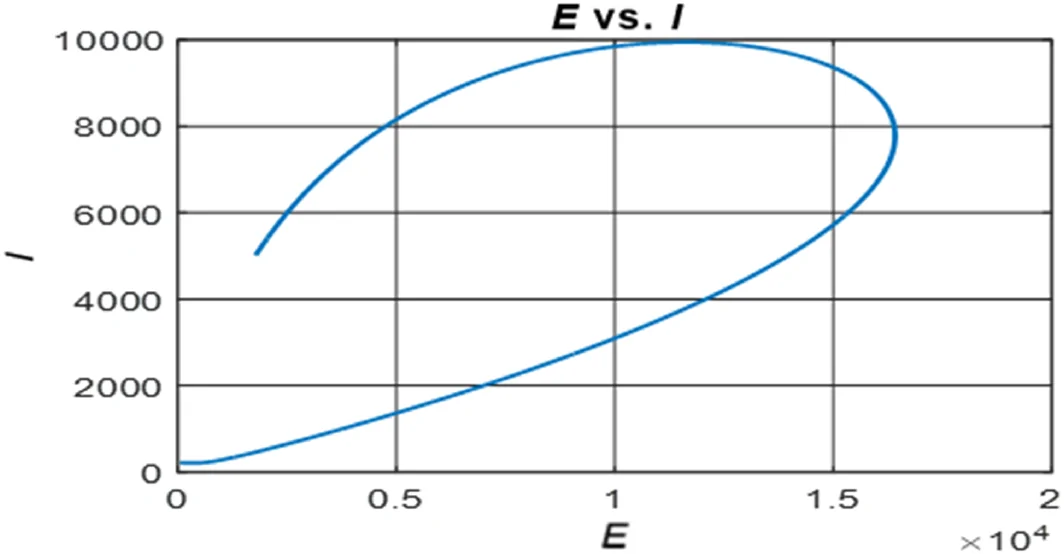
Phase plane of exposed and aware population dynamics.

**FIGURE 12:**
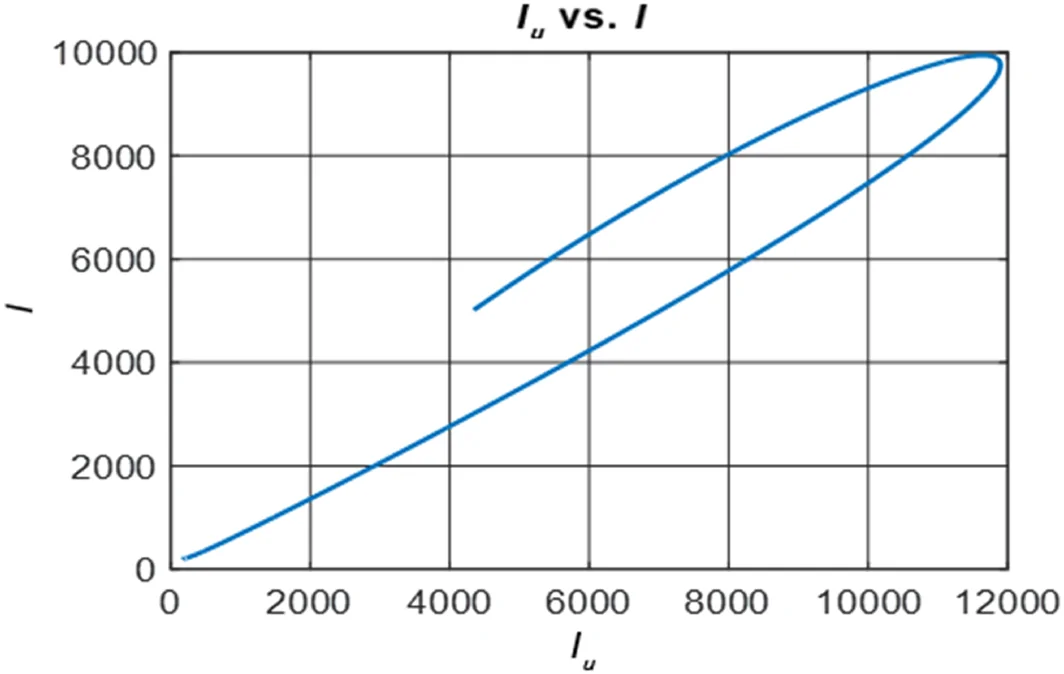
Phase plane of unaware and aware population dynamics.

**FIGURE 13:**
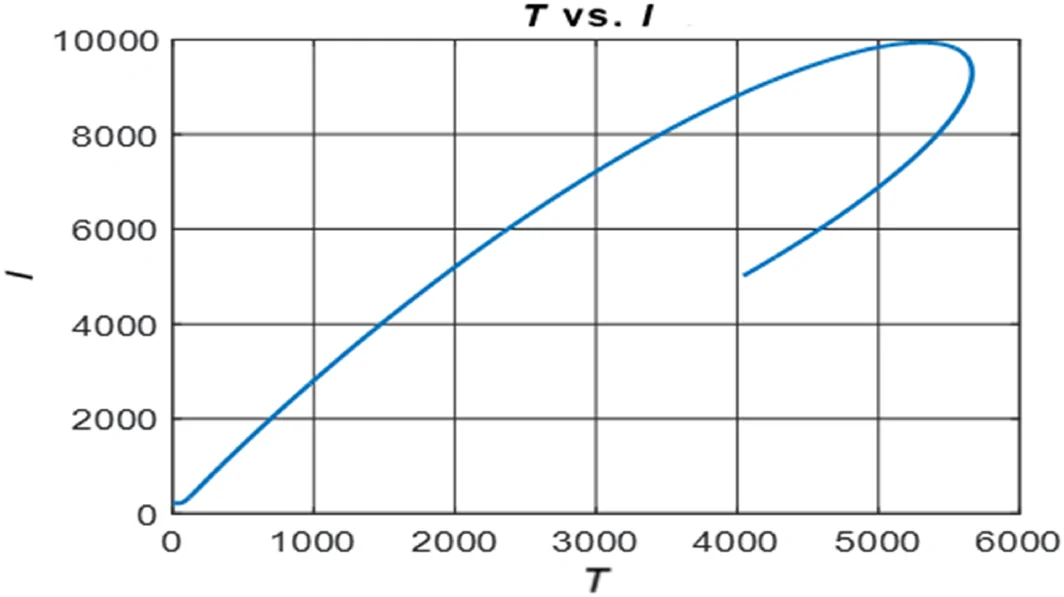
Phase plane of on treatment and aware population dynamics.

**FIGURE 14:**
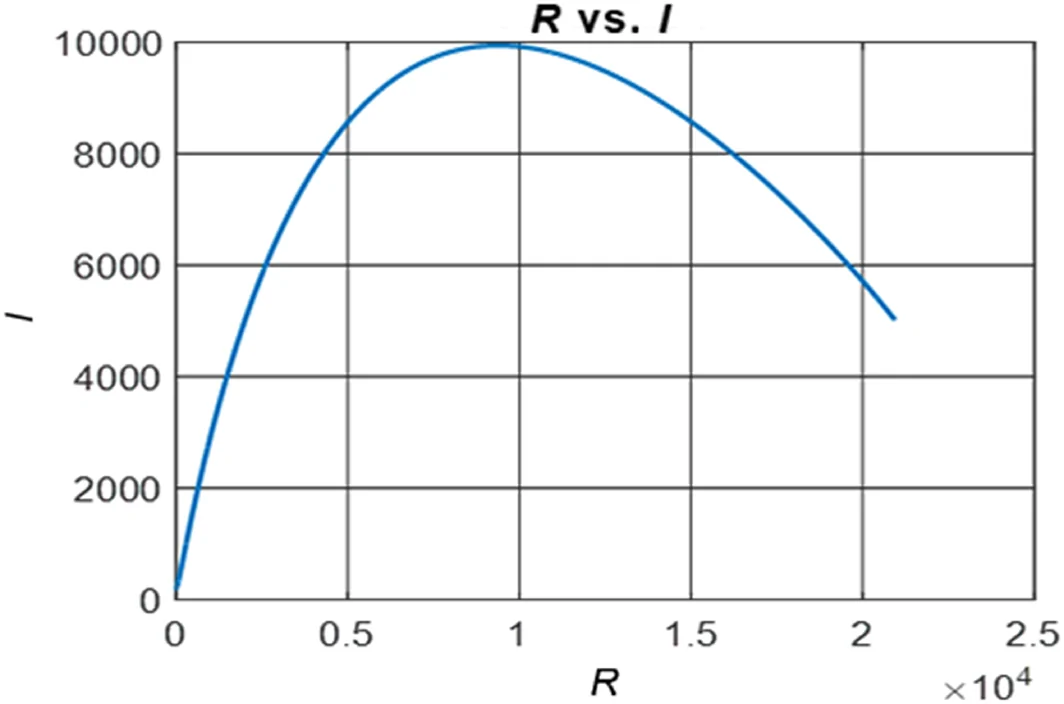
Phase plane of recovered and aware population dynamics.

**FIGURE 15:**
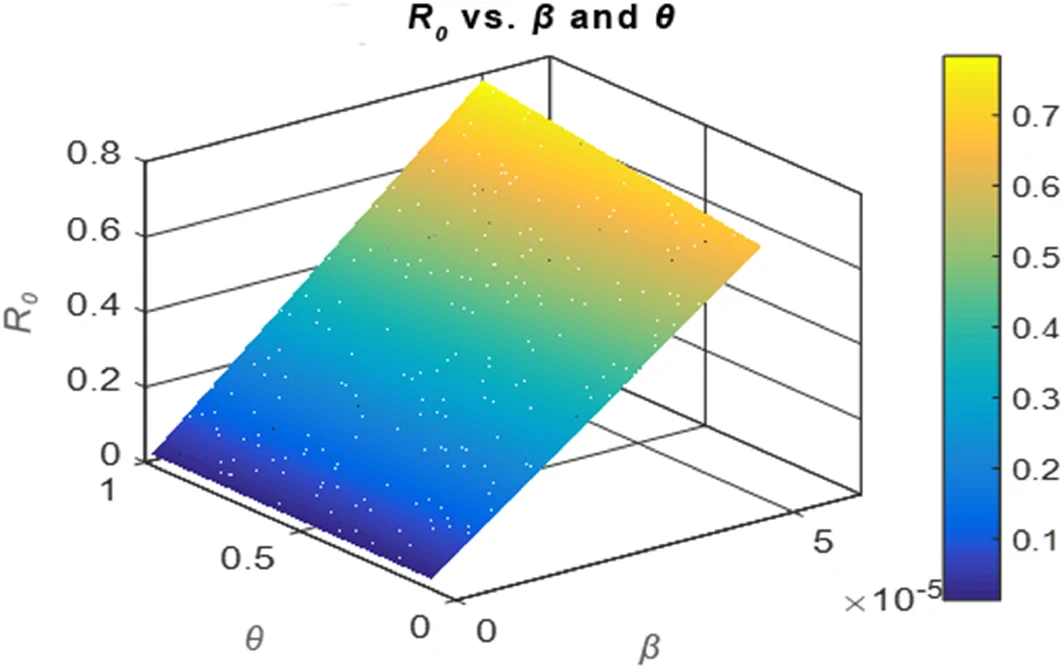
Basic reproduction number illustration for transmission rate and modification parameter.

## 3 DISCUSSION

This section provides an interpretation of the results of the suggested mathematical model for fractional‐order TB transmission. The discussion of results entails the model fitting, sensitivity analysis, reproduction number determination, fractional order dynamics, and phase plane dynamics, where every figure is analyzed in light of the epidemiological model. Figure 1 depicts the data on the TB incidence in relation to the model predictions of incidence from 2000 to 2023. From the results, it can be noted that there is good agreement between the model predictions and the observed data, hence suggesting that the estimated parameters effectively model the transmission dynamics of the disease. The figure below provides an illustration of the local sensitivity indices for the basic reproduction number $R_{0}$ with respect to the model parameters. As can be seen from the results, β and Λ have the highest positive indices which imply that they contribute significantly in increasing $R_{0}$ and promoting transmission of the infection.

On the other hand, parameters like μ,δ, and ω have negative sensitivity indices which imply that they contribute to the decrease in transmission and control of the disease. The figure below also represents the PRCC global sensitivity indices analysis for the basic reproduction number $R_{0}$. As expected from the local analysis, β and Λ have very high positive correlations with $R_{0}$ while μ,δ, and ω exhibit strong negative correlations.

In Figure 4, we present the behavior of the susceptible individuals with respect to time. It can be seen that the number of susceptible is decreasing in time due to the propagation of disease. Fractional order leads to slower decrease as memory effect reduces the rate of infection. In Figure 5, the behavior of the exposed group is shown. Initially, the exposed group rises with an increase in infection rates. Then it starts declining with an advancement towards either infectious or another compartment. Smaller values for *α* result in more persistence of the exposed group, showing delayed progression of TB infection. Figure 6 demonstrates the dynamics of the unreported infectious $I_{u}$. The obtained findings clearly demonstrate the effect of the fractional order on the magnitude of the unreported infections. It can be stated that a higher level of fractional order significantly decreases their occurrence and makes them appear later. Unreported infection plays a crucial role in TB transmission within the population. In Figure 7 the dynamics of active infection is presented.

The changes in the treatment population can be viewed in Figure 8. In view of the outcomes presented above, the number of treatments grows with increasing diagnosis and inclusion of patients into the process. However, a smaller fraction order leads to lower growth rates due to delays associated with the detection and treatment process. The change in the recovered population is depicted in Figure 9. As is seen from the outcomes provided above, recovery steadily increases because of treatment procedures performed. Recovery is characterized by delayed growth rates due to memory effects impacting disease development and treatment.

Figure 10 depicts the phase plane for susceptible S and infectious I populations. As can be seen from the outcome obtained, an increase in infection rate leads to a decrease in the susceptible population. This clearly indicates that the transmission of TB infection takes place as was expected from the outset. The relationship between the exposed E and infectious I populations is illustrated in Figure 11. As is evident, there is a smooth passage from latent infection to active disease state indicating stable and biological consistent dynamics. Phase plane between I and $I_{u}$ populations is shown in Figure 12. The simulation shows both continue increasing for a while and both decrease after a long period. Hence, an unaware and aware population should be considered to control the dynamics of TB infection in Ethiopia. Phase plane between T and I populations is shown in Figure 13. It is evident from the outcomes that an increase in *T* results in a decrease in *I*, thereby highlighting the role of the effectiveness of treatment intervention in disease control. Phase plane between R and I is provided in Figure 14. It is noted that an increase in recovery occurs when infection is minimized over time. Phase plane between $R_{0}$ and the parameters β and θ is shown in Figure 15. It is observed that an increase in either of the two parameters causes an increase in $R_{0}$; hence the significance of these transmission parameters in disease persistence.

Finally, it is evident that the fractional‐order TB model proposed gives an effective way of modeling and studying TB dynamics. In addition, it has been observed that the use of memory effects in the model significantly enhances disease modeling, and sensitivity and phase plane analysis reveal the significant disease control parameters.

## 4 CONCLUSION

In this manuscript, a fractional‐order $SEI_{u}ITR$ mathematical model was proposed and used to explore the transmission of TB. The basic properties of the model, including the existence, positivity, and boundedness of solutions, were proved to guarantee the feasibility of the model. The basic reproduction number $R_{0}$ was calculated and used to establish the threshold condition for disease persistence and elimination. The model was successfully used to fit the TB incidence data from Ethiopia, and it was found that there was a good agreement between the model and real data, which confirmed the accuracy and reliability of the model. The sensitivity analysis was carried out and showed that the transmission rate β and the modification parameter θ are directly proportional to $R_{0}$ and play a significant role in disease transmission, and the parameter ω associated with intervention is the most sensitive parameter in reducing $R_{0}$ and thus is significant in disease control through strategies such as early diagnosis, treatment, and awareness. Additionally, the fractional‐order analysis showed that increasing the fractional‐order decreases the level of infection and improves stability, as a result of the memory effect included in the fractional derivative. Stability of the DFE was proved for $R_{0}<1$, which confirms that TB can be controlled if appropriate intervention strategies are put in place. Future work can be carried out by adding other epidemiological parameters, which include vaccination, reinfection, and drug resistance, and developing optimal control strategies to determine how to control TB. Stochastic, spatial, and co‐infection factors, which include TB and lung cancer progression, can be included to make the model more realistic. This can be done in future work to make the model more applicable in supporting public health decisions and improving TB control strategies.

## 5 METHODS

The current fractional model TB transmission consists of

1. Susceptible (S): This compartment encloses TB free individuals with a chance to be infected in the future.
2. Exposed (E): This class includes persons who have been in contact with the TB bacterium but are not yet infectious and thus cannot spread the disease.
3. Undetected (unaware) infected ($I_{u}$): This compartment represents persons who are infected with TB but are not yet aware of their status.
4. Detected (aware) infected (I): This class comprises persons who are TB positive but not on treatment.
5. On treatment (T): This compartment includes persons who are receiving treatment for TB infection.
6. Recovered (R): This class comprises persons recovered.

The total population size at time *t*, represented by N(t), is expressed as

(117) $$N\left(t\right)=S\left(t\right)+E\left(t\right)+I_{u}\left(t\right)+I\left(t\right)+T\left(t\right)+R\left(t\right).$$

The explanation of the state variables and parameters used in the model development is presented in Tables 4 and 5, respectively.

**TABLE 4:** Notation and description of state variables in the TB model.

State variable	Description
S(t)	Number of susceptible individuals at time *t*
E(t)	Number of exposed individuals at time *t*
$I_{u}\left(t\right)$	Number of TB‐infected but unaware individuals at time *t*
I(t)	Number of TB‐infected and aware individuals at time *t*
T(t)	Number of individuals undergoing TB treatment at time *t*
R(t)	Number of recovered individuals at time *t*

Abbreviation: TB, tuberculosis.

**TABLE 5:** Notation and description of parameters in the TB model.

Notation of parameter	Description of parameter in TB model
Λ	Recruitment rate into the susceptible population
β	Transmission rate of TB infection
θ	Modification parameter for TB transmission from aware infected individuals (I)
σ	Modification parameter for TB transmission from treated individuals (T)
κ	Transfer rate from exposed (E) to unaware infected ($I_{u}$)
ω	Transfer rate from exposed (E) to aware infected (I)
γ	Transfer rate from exposed (E) to treatment class (T)
$\delta_{1}$	TB‐induced death rate of unaware infected individuals ($I_{u}$)
$\delta_{2}$	TB‐induced death rate of aware infected individuals (I)
$\delta_{3}$	TB‐induced death rate of individuals under treatment (T)
ϕ	Treatment rate of unaware infected individuals ($I_{u}$)
ρ	Treatment rate of aware infected individuals (I)
η	Rate of TB recovery
μ	Common biological death rate
α	Order of the fractional derivative, 0<α≤1

Abbreviation: TB, tuberculosis.

In the construction of the schematic diagram of the TB dynamic transmission system, it is assumed that the disease induced death rate $\delta_{1}=\delta_{2}=\delta_{3}=\delta$. The fractional dynamics of each variable on Figure 16 TB model is given by

(118) Dtα0CFS(t)=Λ−βS(t)Iu(t)+θI(t)−μS(t),Dtα0CFE(t)=βS(t)Iu(t)+θI(t)−(ω+κ+μ)E(t),Dtα0CFIu(t)=κE(t)−(ϕ+δ+μ)Iu(t),Dtα0CFI(t)=ϕIu(t)+ωE(t)−(ρ+δ+μ)I(t),Dtα0CFT(t)=ρI(t)−(η+δ+μ)T(t),Dtα0CFR(t)=ηT(t)−μR(t),

with initial conditions

(119) $$S\left(0\right)\geq 0,E\left(0\right)\geq 0,I_{u}\left(0\right)\geq 0,I\left(0\right)\geq 0,T\left(0\right)\geq 0,R\left(0\right)\geq 0.$$

**FIGURE 16:**
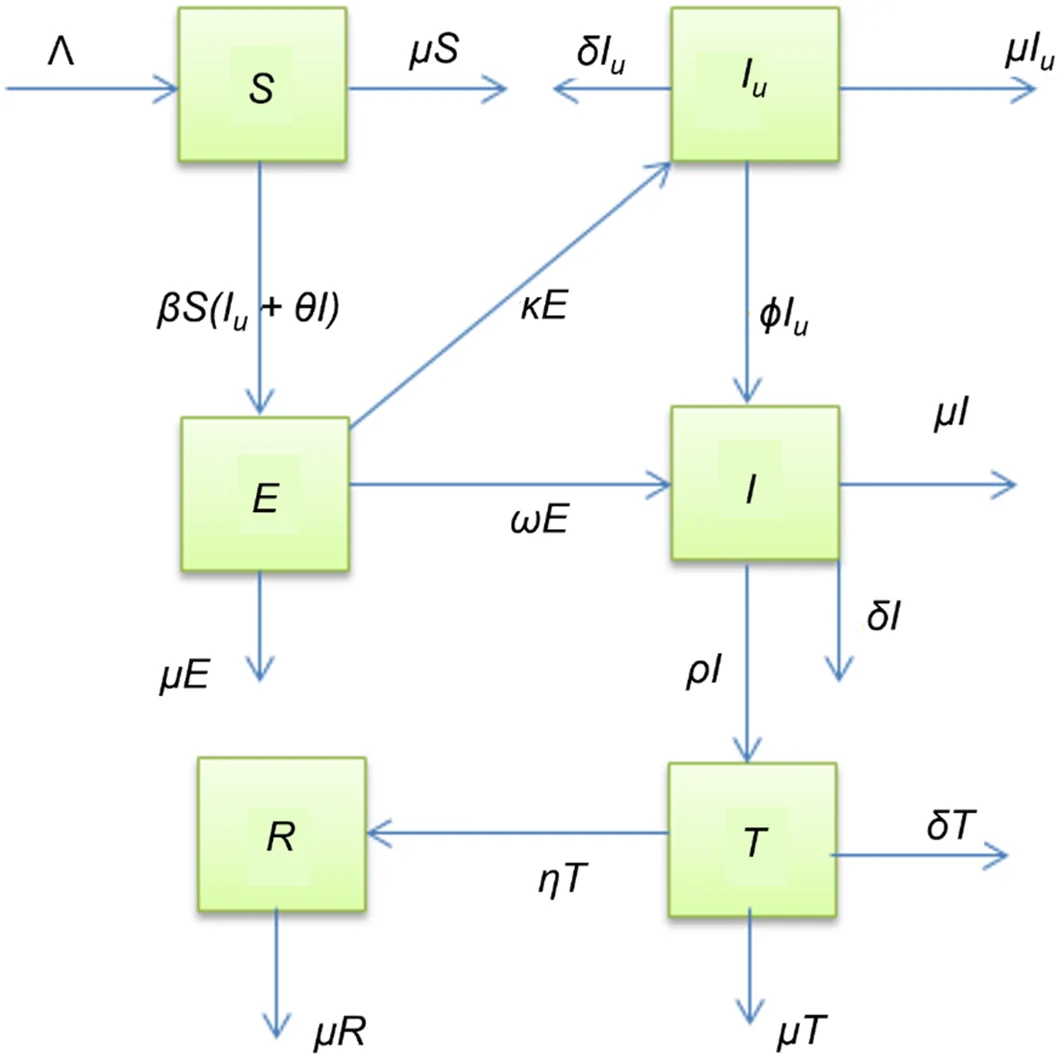
Tuberculosis schematic diagram.

## AUTHOR CONTRIBUTIONS

**Kumama Regassa Cheneke**: Conceptualization; formal analysis; methodology; software; writing—original draft. **Fatmawati Fatmawati**: Supervision; validation; writing—review and editing. **Mustafa Bayram**: Writing—review and editing; validation.

## CONFLICT OF INTEREST STATEMENT

The authors declare no conflicts of interest.

## ETHICS STATEMENT

This study does not involve human participants, animal research, or the use of personally identifiable data.

## Data Availability

The numerical data are included in the manuscript.
